# Seroprevalence and Risk Factors of *Chlamydia* Infection in Pigs in Hunan Province, Southern China, 2017–2018

**DOI:** 10.1089/vbz.2023.0064

**Published:** 2024-04-08

**Authors:** Junkun Yang, Shilin Chen, Minxiu Quan, Leqin Li, Ling Shang, Zhongxin Fan, Shifeng Hu

**Affiliations:** ^1^ College of Veterinary Medicine, Hunan Agricultural University, Changsha, PR China.; ^2^ Central South University of Forestry and Technology, Changsha, PR China.; ^3^ Wanning Animal Disease Prevention and Control Center of Hainan Province, Wanning, PR China.; ^4^ Animal Disease Prevention and Control Center of Hunan Province, Changsha, PR China.

**Keywords:** *Chlamydia*, Hunan Province, risk factors, seroprevalence, pigs

## Abstract

**Background:**

*Chlamydia* is a Gram-negative obligate intracellular bacterium that is pathogenic for humans and a large variety of veterinary animal species. However, there is no continuous monitoring of *chlamydia* infection data in pigs in Hunan province, southern China. Therefore, in order to evaluate the seroprevalence and identify risk factors associated with *Chlamydia* infection in pigs within this region, a comprehensive study was conducted.

**Methods:**

A total of 3848 serum samples were collected from pigs (from farmers and companies) between May 2017 and August 2018. The presence of specific antibodies against *Chlamydia* was determined through the employment of the indirect hemagglutination assay (IHA).

**Results:**

The overall seroprevalence of *Chlamydia* was determined to be 26.90% (1038/3848, 95% confidence interval: 25.60–28.40). By employing statistical analysis using SPSS software (*p* < 0.05), factors such as altitude, sampling regions, and rearing systems of pigs were identified as potential risk factors for *Chlamydia* infection.

**Conclusion:**

These findings elucidate a substantial prevalence of *Chlamydia* in pigs within the mountainous region of Hunan province, southern China, thereby highlighting a potential risk to human health. These results underscore the need for proactive measures and targeted interventions to mitigate the transmission of Chlamydia in porcine populations, safeguarding both animal welfare and public health.

## Introduction

*C**hlamydia* is an obligate intracellular pathogenic microorganism, Gram-stain-negative, and has a two-phase life and development cycle of the primary body and reticulate body. It exhibits a global distribution and infects a broad spectrum of hosts, ranging from protists to higher vertebrates, encompassing amoebas, insects, aquatic animals, reptiles, avians, mammals, and humans. Notably, there is currently no effective vaccine available to combat *Chlamydia* infections (Elwell et al., 2016, Horn, 2008, Schautteet and Vanrompay, 2011). At present, there are 2 genera of *chlamydia* in the *chlamydia* family, encompassing a total of 20 identified members, some of which, such as C. *abortus*, C. *pecorum*, and C. *suis*, have the ability to infect pigs (Laroucau et al., 2020; Laroucau et al., 2019; Staub et al., 2018; Taylor-Brown et al., 2017; Taylor-Brown et al., 2016; Vorimore et al., 2021; Vorimore et al., 2013). Several studies have documented a high prevalence of mixed infections with C. *suis* and C. *abortus* in the pulmonary and gastrointestinal tracts of pigs (Hoelzle et al., 2000; Rohner et al., 2021).

Moreover, reports from China have indicated varying prevalences of chlamydial infections, ranging from 18.79% in piglets to 80.89% in pregnant sows (Jiang et al., 2013; Li et al., 2017; Nie et al., 2018; Sun et al., 2020; Zhang et al., 2014). Previous research has suggested the zoonotic potential of *C. suis*, as it has been identified in single or mixed infections with *C. trachomatis* (Dean et al., 2013). In addition, *C. suis* DNA has been detected in conjunctival swabs of employees in a Belgian pig slaughterhouse, as well as in pharyngeal and rectal swabs of Belgian pig farmers, highlighting the potential public health implications of pig pathogens (De Puysseleyr et al., 2017; De Puysseleyr et al., 2014). Consequently, pig pathogens may not only affect the pig production, but also potentially play a role in public health.

China, being a predominantly agricultural nation, relies heavily on animal husbandry, with pork accounting for over 50% of domestic meat consumption in 2022 (source: https://www.statista.com/). The country holds the distinction of being the world's largest pork producer, consumer, and importer. Chlamydial infection in pigs has been reported in various provinces of China, such as Shandong (Sun et al., 2020), Yunnan (Bi et al., 2011), Fujian (Zhou et al., 2008), Jiangxi (Jiang et al., 2013), and Hunan (Zhang et al., 2014) provinces. However, the majority of these studies have been published in local journals in the Chinese language, making them less accessible to international researchers. Furthermore, despite being the second largest pig producer in China, there is a lack of continuous epidemiological reporting on chlamydial infections in pigs in Hunan province, hindering the monitoring and management of *Chlamydia*.

The aim of this study is to investigate the seroprevalence of *Chlamydia* in pigs within Hunan province and explore the underlying factors contributing to the epidemic of *Chlamydia*. The findings of this research endeavor will provide valuable data to enhance precautionary measures and control strategies aimed at preventing *Chlamydia* infections in pigs across China.

## Materials and Methods

### Investigation sites

**FIG. 1.**
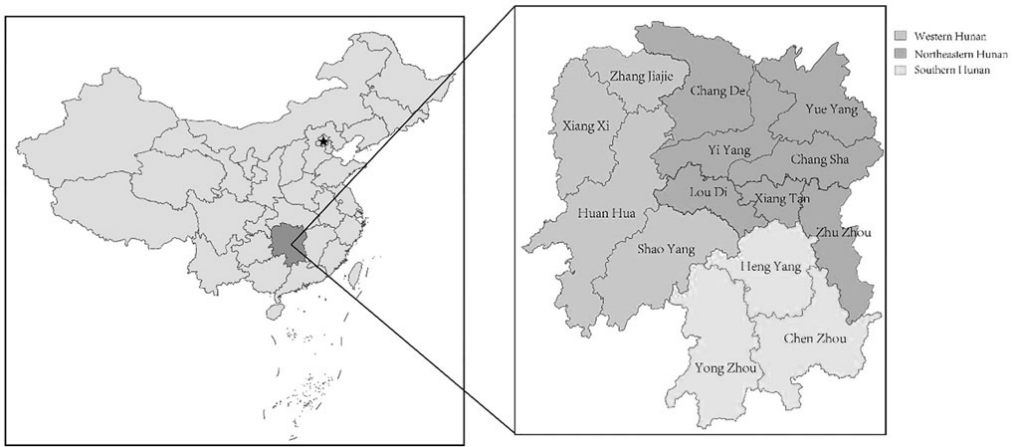
Map showing the geographical locations and seroepidemiological distribution of Chlamydia in Hunan Province, China.

This study involved 14 regions throughout Hunan Province, south China (Fig. 1), including 7 cities located in Northeastern Hunan, 4 cities located in Western Hunan, and 3 cities located in Southern Hunan, respectively. Hunan Province (24°38′–30°08′ N, 108°47′–114°15′ E) is located in the south of China, which has a subtropical monsoon climate with distinct differences in the four seasons. Additionally, from east to west, the altitude gradually decreases. The western and southern Hunan are dominated by mountains, while the northern Hunan is dominated by lakes, resulting in a significant difference in weather.

### The collection of serum samples

Three thousand eight hundred forty-eight serum samples were randomly collected from pigs from 14 regions in Hunan Province between May 2017 and August 2018 (Table 1), including Heng Yang City (*n* = 106), Yong Zhou City (*n* = 221), Chen Zhou City (*n* = 543), Chang De City (*n* = 804), Yue Yang City (*n* = 205), Yi Yang City (*n* = 121), Chang Sha City (*n* = 549), Lou Di City (*n* = 298), Xiang Tan City (*n* = 327), Zhu Zhou City (*n* = 339), Zhang Jiajie City (*n* = 64), Xiang Xi City (*n* = 120), Huai Hua City (*n* = 60), and Shao Yang City (*n* = 91); with 870, 2643, and 335 serum samples being collected from Southern Hunan, Northeastern Hunan, and Western Hunan, respectively. The serum samples were transported to the laboratory and stored at -20°C.

**Table 1.** The Seroprevalence of *Chlamydia* Infection in Pigs in Different Cities of Hunan Province, China

Region	City	No. examined	No. positive	Prevalence (%)
Southern Hunan	Hengyang	106	46	43.40
	Yongzhou	221	88	39.82
	Chenzhou	543	89	16.39
	Total	870	223	25.63
Northeastern Hunan	Changde	804	223	27.74
	Yueyang	205	5	2.44
	Yiyang	121	28	23.14
	Changsha	549	195	35.52
	Loudi	298	119	39.93
	Xiangtan	327	93	28.44
	Zhuzhou	339	89	26.25
	Total	2643	752	28.45
Western Hunan	Zhangjiajie	64	11	17.19
	Xiangxi	120	7	5.83
	Huaihua	60	37	61.67
	Shaoyang	91	8	8.79
	Total	335	63	18.81
Total		3848	1038	26.90

### Serological tests

The specific antibodies against *Chlamydia* at genus level from pigs were detected by indirect hemagglutination assay (IHA) using a commercially available kit (Lanzhou Veterinary Research Institute, Chinese Academy of Agricultural Sciences, Lanzhou, China) according to the manufacturer's instructions, and this kit can be used to detect antibodies against *Chlamydia* at the genus level in all mammals (Li et al., 2020, Ni et al., 2015, Zhang et al., 2014). Briefly, the serum was diluted by serial fourfold from 1:4 to 1:64, and the positive and negative serum were separately added to each plate. The serum samples were considered positive if agglutinated layers of erythrocytes formed in a dilution of 1:16 or higher. The results between 1:4 and 1:16 were considered as “doubtful” and were retested.

### Statistical analyses

Differences in the seroprevalence of *Chlamydia* in pigs of different geographical origins and categories were analyzed with *T* test by using SPSS software (SPSS, Inc.; version 19). The cutoff for statistical significance was *p* < 0.05.

## Results and Discussion

In this study, the presence of antibodies against *Chlamydia* was detected in 1038 out of 3848 randomly selected pig serum samples, resulting in a positivity rate of 26.90% (95% CI = 25.60–28.40) using the IHA with a 1:16 cutoff. Comparative analysis with previous research findings (Tables 1 and 2) reveals a gradual upward trend in the prevalence of chlamydial infection in Hunan Province as a whole. Several factors contribute to this observation:

**Table 2.** The Seroprevalence of *Chlamydia* Infection in Pigs in Different Regions of China

Region	Prevalence (%)	Date of investigation	Method	Literature
Hunan Province	9.00	1986.04–1987.12	IHA	Jiang et al. (1989)
	16.70	2000.08	IHA	Qiu et al. (2003)
	22.25	2004.03–2005.04	IHA	Li (2005)
	62.70	2010.01–2012.08	IHA	Zhang et al. (2014)
Tibet Province	16.63	2010.04–2010.12	IHA	Zhang et al. (2013)
Jiangxi Province	58.59	2012.01–2012.12	IHA	Jiang et al. (2013)
Fujian, Jiangsu, Zhejiang Province, etc.	62.40	2015–2016	FRET-PCR	Li et al. (2017)
Shandong Province	24.15	2017.01–2018.12	IHA	Sun et al. (2020)
Yunan Province	18.50	NA	IHA	Bi et al. (2011)

FRET-PCR, Fluorescence Resonance Energy Transfer-PCR; IHA, Indirect Hemagglutination Assay; NA, not applicable.

(1) Before 2000, the pig industry predominantly comprised individual farmers operating on a small scale (three to five pigs). These farmers were less inclined to proactively report pig health issues. Even in cases of pig illness or mortality, they would typically slaughter and consume the animals themselves, resulting in potentially unreliable data.
(2) Since 2014, the pig industry has shifted toward corporate dominance, leading to improved standardization and increased attention to infectious diseases. Consequently, data collection, monitoring, and traceability have become more sufficient.
(3) Historically, pig breeding and sales were largely confined to individual cities or provinces, limiting the spread of epidemics (−2000). However, the modern pig industry spans multiple provinces, facilitating rapid transmission of diseases once outbreaks occur, as was witnessed during the African swine fever outbreak in 2018.

Comparison with other regions (Tables 1 and 2) reveals that the prevalence of mycoplasma infections is relatively lower in the mountainous western region, while the plains and lakes of the north and central regions exhibit a relatively higher prevalence. Specifically, Tibet and Yunnan (western China with mountainous regions) exhibit prevalence rates of 16.50% and 18.80%, respectively, which are lower than the overall level observed in Hunan Province. Conversely, Jiangsu (eastern China with lakes) and Shandong (northern China with plains) exhibit higher prevalence rates compared with Hunan Province. Moreover, within Hunan Province, the prevalence of chlamydial infections is lower in the mountainous western areas (Xiangxi, Shaoyang, Zhangjiajie) compared with the northern plains. These findings suggest the need for targeted regional divisions to facilitate focused prevalence assessment, prevention, and control strategies.

The findings of this study reveal that pigs in the western region of Hunan had the lowest susceptibility to *Chlamydia* infection. This observation may be attributed to the unique topography of Hunan Province, where the southern and western regions are characterized by mountainous terrain, while the northeastern part of the province boasts abundant water resources and expansive areas. These geographical variations, coupled with a higher density of live pigs in certain areas, likely contribute to the differences in *Chlamydia* seroprevalence.

It is important to note that the data collected in this study had limitations regarding the information obtained from farmers and companies. To enhance the understanding of *Chlamydia* infection risk factors in pigs in China, more comprehensive and detailed information is necessary. This includes factors such as gender, age, breed, sources of water and food, as well as other potential exposures that pigs may encounter, and the specific conditions of their rearing environments. Future studies should aim to investigate these specific elements to refine our knowledge of the risk factors associated with *Chlamydia* infection in pigs and improve the accuracy of the data.

## Conclusions

In conclusion, the results of this study indicated that *Chlamydia* seroprevalence of pigs is quite high in the mountainous region of Hunan province, southern China. Comparative analysis with previous research findings (Table 2) reveals a gradual upward trend in the prevalence of chlamydial infection in Hunan Province as a whole. Moreover, sampling regions, and rearing system were identified as risk factors for *Chlamydia* infection in Hunan province. For example, the prevalence of chlamydial infections is lower in the mountainous western areas (Xiangxi, Shaoyang, Zhangjiajie) compared with the northern plains. The findings of this study reveal that pigs in the western region of Hunan had the lowest susceptibility to *Chlamydia* infection. Our results could provide important data for the strategic control and prevention of *Chlamydia* infection in China.

## Ethics Approval and Consent to Participate

All experimental procedures involving animals complied with the National Research Council's Guide for the Care and Use of Laboratory Animals and were approved and overseen by the Institutional Animal Care and Use Committee at Hunan Agricultural University (no. 2021085).
